# Crime and Punishment

**Published:** 1913-08-02

**Authors:** 


### CRIME AND PUNISHMENT.

The Section of Medical Sociology of the B.M.A., held at Brighton, under the presidency of Dr. Reginald J. Ryle, J.P., discussed at some length, from the Medical, Legal, and Social Aspects, the Subject of Crime and Punishment.

The President

Pointed out that this Section differed from all the others in the Association in that it was not entirely composed of medical men, but invitations were offered to legal and lay members of the public to help in the reading of papers and discussions on subjects rather communal than medical. To-day the solemn and serious problems connected with crime and punishment were to be dealt with. It was felt that the medical world should have a larger basis than what was implied in the respective terms "criminal mind" and "criminal act." In other branches knowledge had progressed by the splitting up of subjects into sub-divisions, each of which was studied specially. It was not so easy now as a century ago to decide who was and who was not responsible and who was criminal. Thus it was not surprising to find on the statement of the author of "Ancient Law" that our theories of crime and punishment had broken down, and that we were thus necessarily thrown back more and more upon experience; and it was by the discussion of experience that progress would be made in connection with this particular subject.

### Crime and Criminals.

Dr. James Scott (late Governor of H.M. Prison, Holloway),

In a careful paper, considered that few subjects were more difficult than that of crime and how to deal with criminals, and there had naturally been a very extensive literature on the subject, and there was still room for further debate. Crime was an offence against social order, and in the interests of the community it was visited with punishment. The view was held by some that crime was a disease, and that criminals should be pitied and not punished, and something could be said for that view. As with disease, so with crime; some subjects of it could act as foci of infection to others and so must need isolated treatment. In connection with both prevention was better than cure. Every effort should be made to improve social conditions, but society must protect itself by dealing with criminals in some way. Carlyle had written a scathing passage condemnatory of the "benevolent platform fever" and "beneficent sentimentality." A healthy public opinion which deprecated crime was a potent factor making for improvement. A bishop had recently deplored a national decline in the sense of responsibility. In early days the motive of punishment seemed to have been vengeance; it was felt that the person punished owed some expiation to society for having broken its laws. But the real purpose of punishment was to protect society and to deter persons similarly disposed from committing a like act. There was now prevalent a strong desire to do all that was reasonable to reclaim the criminal by bringing good influences to bear upon him. On the question of who should be punished, children under seven and practically under fourteen, were presumed by the law to be incapable of crime. It had been urged that there should be medical assessors at trials, but there were difficulties in the way. Every care was taken to avoid doing an injustice: if the prisoner were too intoxicated to be able to reason about his acts that was remembered in mitigation. With the spread of education there had been fewer cases of offences against the person, but there was an increase in the number of criminal frauds, for which science was largely employed. But the police also did their best to enlist science on their side, and they were often unjustly criticised. Finger-prints and classification were largely employed, and the recent refined method of taking and examining the pores and orifices of the sweat glands was said to offer great help. The author proceeded to deal with the question of the abolition of capital punishment, and the substitution for treadmills of industrial labour, as well as the improved diet and hygienic conditions in prisons. The "Borstal" system had done excellent work in reference to the young offender, and was likely to do even more in the future. Much was to be said in favour of consideration of the criminal himself rather than the crime itself, for one was then able to deal more justly.

### The Genesis of Crime.

Dr. Charles A. Mercier's

Paper was concerned with the genesis of crime, whether it was a biological genesis or otherwise. Crime, he said, was a consequence of the social state. A person living in solitude could not commit a crime any more than a man in an arid desert could drown himself. Though it had long been felt that crime was an offence against society, it needed much amplification and interpretation to make it express the whole truth. Kant advised us so to act that our conduct might be a law to all men, from which it could be concluded that it was wrong to do that which, if everyone did it, would be detrimental or destructive to society. Wrongful acts could be divided into those of greater and those of lesser gravity, according to whether they were destructive of or merely detrimental to society. If homicide or theft were practised by everyone on all available occasions, social life would be impossible, and man must become solitary. If everyone allowed his chimney to catch fire, or drove in the dark without a light, society would not be broken up, but would suffer manifest disadvantage or detriment. A crime or wrongful act must harm someone, otherwise it was neither a crime nor wrongful. Thus one returned to the original thesis that crime was a consequence of the social state. An act must be injurious to society by striking at its base, its representative, or its fabric, or indirectly by striking not at society as a whole, but at some member or members of society, so tending to break society up piecemeal by a process of attrition. Or it might strike at the racial principle by diminishing the supply of future members to take the place of those who must, in the course of nature, perish. In saying that, he did not conflict with what he had stated elsewhere, that crime was that which was punishable by law, for both were true. Law was founded on the principle of social preservation. Though law-makers had not that principle articulately or formally before their minds when the laws were made, the basis of their work was an instinctive appreciation that certain acts were antagonistic to the social principle, and if permitted to prevail would be destructive of society. Without the machinery for maintaining the fabric of society and suppressing assaults and injuries to society as a whole, society as at present constituted would dissolve in anarchy. For people to live together in society they must have some motive to come together and to keep together; and this arose from an inborn desire for the society of one's fellows and an aversion to continued solitude. For the welfare of society it was much less important that laws should be good than it was that laws should be observed. Hundreds of communities had survived under bad laws which were enforced, but no community had long existed where laws were not enforced. If the principle of the destruction of society by crime was recognised, what was the origin of crime? Other animals, such as bees, ants, and wasps, lived under equitable and harmonious conditions and made greater sacrifices: why did not man? He believed it was because, comparatively speaking, man had been socialised as yet for but a brief period, and hence his system was less perfect; selfish instincts and desires were less subdued, and when there was a conflict with social instincts, the victory was sometimes gained by selfishness, and then the result was crime. The root of crime was the determination to obtain gratification for oneself regardless of the cost to others. Treason was in a class by itself, and it meant an attack on the fabric of society or on the governing body; but anarchy was a refusal to obey law as law, not an objection to any particular law on the score of it being unjust. That was the gravest of all crimes, because it was most subversive of the social fabric. Society was bound to suppress it at any cost. There was a small class of crime which was looked upon with abhorrence and was visited with severity, though such crimes might injure no one, and might seem at first not to concern anyone. He referred to crimes of sexual perversion—rape, the procuring of abortion, and such like. They offended against the racial principle; they prevented others coming into the world to take the place of those who were becoming old and so losing their social value. Abhorrence of these offences indicated a valuable and healthy instinct. It was the imperious instinct of every man that crime should be suppressed. If selfishness was at the basis of crime, then if it could be shown to the potential criminal that it was against his own interest to commit crime, then that very ruling principle could be used to cause him to do for his own sake that which he would not have done for the sake of society. In other words, the efforts should be directed to showing the criminal that crime did not pay. But if he were told he was not a scoundrel, but a misunderstood unfortunate, one must not be surprised if statistics of crime increased.

### THE MEANING AND USE OF STATE PUNISHMENT.

Sir H. Bryan Donkin,

In an elaborate paper, dealt with the meaning and use of State punishment. He referred to the recent literature which set out to assert that the State ought not to punish the criminal, and in various ways arrived at the conclusion that crime was the result of a vicious system. Mr. Tighe Hopkins wrote that State imprisonment as a means of punishment had failed, yet he had to admit that garrotting was checked by the application of the ordinary law and ordinary punishments. A criminal who spent his intervals in reading literature dealing with these questions told the author that he felt persuaded that he had inherited his instincts, and was not personally responsible. It had also been said that society, not the criminal, was responsible for crime. In the paper he assumed certain postulates: (1) That the State was justified in coercing law-breakers with a view to the protection of the community. This justification was established even if such coercion were expressly directed only to the protection of the community and the deterrence of others. Punishment meant the infliction of pain and suffering, no matter what the avowed object of it might be. The mere loss of liberty was a definite infliction of suffering, although it might be ordered for the protection of society. Prisons, even if they stood empty, must constitute deterrents to crime. He did no propose to discuss the question whether the criminal was born or made; whatever the antecedents of a man might be, every man was very largely made. His second postulate was that not only the harm done to society, but also, as far as practicable, the character and condition of the criminal must be taken into account in correction and treatment. (3) The controversy concerning

"free will" and "determinism" did not concern the practical penologist. (4) All men were potential law-breakers, even as they were tempted to put their own affairs before the welfare of society. He did not believe there was any "criminal type," and he considered that the formulated doctrines of "hereditary crime" were misleading. Even if the object were reformation, the element of retaliation was inevitable in the use of coercion. Persons of defective mind, especially habitual criminals, should be carefully investigated, and they should not be punished more than was necessary for the social object in view; frequently recurring sentences of penal servitude were out of place for such. They should be certified as being not normally responsible, and in this connection the French term "attenuated responsibility" was a good one. To take the view of the eugenists that the State must be parental in the full sense, and aim at the prevention of misdoings, was rushing in where thoughtful individuals might well hesitate to tread, but he agreed that every opportunity should be taken to reform by training all potentially reformable offenders, in the way now adopted by the "Borstal" system for young offenders.

### A Board of Punishment.

Mr. A. O. Jennings, LL.B. (Registrar of H.M. High Court of Justice),

Said apparently doctors disclaimed the idea that crime was a disease, and yet he thought that in this meeting, at all events, he would have heard some adherents of that view. The lawyer asked the doctor what forms of this disease should be treated by punishment, for on that matter there had been an unhappy difference among people for sixty years. He had not heard set forth what was the idea of criminal responsibility. Sir James Fitzjames Stephen laid it down that no act was a crime if the person who did it was, at the time it was done, prevented, either by defective mental power or by disease affecting his mind, first from knowing the nature and quality of his act or knowing his act was wrong, or controlling his own conduct unless the absence of power of control had been produced by his own fault. But, said the same authority, an act might be a crime, although the mind of the person who did it was affected by disease if such disease did not produce on his mind one or other of the defects mentioned. He did not see any flaw in the reasoning of that authority, but the difference between the lawyer and the doctor had been as to how far there might be disease affecting the mind which did not affect responsibility. He asked doctors present to say where they thought Sir James Stephen was wrong, because at present the ultimate decision rested with the lawyers. He agreed that our present method of punishment was to a large extent a failure, because the criminal was so often found back in prison again. And one would expect it to fail, seeing that there was little in the system which tended to make the criminal better. One had no assurance that the criminal would be any more fit for society after he had been to prison than before. He did not see why the "Borstal" system should be limited to the young criminal; he would like to see it extended to older prisoners. Was society content to leave decisions to the ordinary jury? And was the Judge the best person to impose the sentence? He thought not, because he knew only so much of the history of the prisoner as happened to be supplied at the trial, and that led him to advocate a Board of Punishment.

### Is Prison a Deterrent?

Rev. C. B. Simpson, M.A. (Chaplain Inspector of H.M. Prisons),

Spoke from an intimate knowledge and personal association with criminals for twenty-five years. To his amazement and amusement, it was now contended that punishment did not deter from crime, but those who wrote in that way had a very limited knowledge of human nature. He had often heard criminals remark "You will not catch me here again." Of 68,000 men first offenders for twelve years, only 6,000 had been re-committed; and of 12,000 women first-offenders during the same years, 1,700 were recommitted, or 9 per cent. and 14 per cent. respectively. One of the chief punishments to the criminal arose from his aversion to solitude. A drunkard committed to seven days was of no use; he simply looked forward to the time when he would slake his thirst again.

Prince Kropotkin

Declared that the prison had not the deterrent effect which the last speaker contended it had. It had been said that the plank-bed and bread and water was a hardship to the prisoner, but it was not so if he had been accustomed to sleeping under bridges, and black bread was not a hardship to those who had had nothing to eat for many days together. Vengeance bred vengeance. He pointed out that in countries which had abolished capital punishment there was no diminution in the deterrent effect of the punishment remaining. He considered that the statistics stated by Mr. Simpson were useless, because there were so many trivial cases, seven days' imprisonment and so on, of persons who were not really criminals at all. He contended that the prison was the university of crime. He had seen children in prison where health was ruined by practices which reduced their capacity to earn their living in the future, or to take their position in society. Interviews with prisoners who were punished for sexual offences showed that they were full of what they would do when they came out. It must be remembered that prison was the place where the physical and mental state was reduced to the minimum. People who had done mathematics were found to be incapable of it after having served a term of imprisonment. Who would receive a prisoner when he or she had been in prison? Only their pals, and there could be no doubt that prisons were anti-social agencies.

### Crime and its Effects on Society.

Capt. A. St. John (Hon. Secretary, Penal Reform League)

Contended that though crime was generally an anti-social act, yet all acts contrary to social order were not crimes. Persons of the most selfish type might never come before the Courts. He referred to a case in which a child was summoned for stealing milk from a doorstep, and the magistrate said the person who left it there should be in the dock. What could be more anti-social than that? People were constantly being put into prison when they should not be there, and hence were being made criminals. There was need for the community to protect itself not only from criminals, but also from the people and the institutions which dealt with criminals; the danger to society done by burglars and thieves in England was a mere bagatelle compared with the harm done by the laws of England. Everybody knew that the Law Courts were constantly putting into prison boys who ought not to be there. To sentence a drunkard to seven days was ineffectual, and if so, it was anti-social. To deal with the criminals, as with the patient, effectively, the first need was to diagnose the case, and that should include careful inquiry into the person's antecedents. Otherwise one could not be sure that injustice was not being done. It was said there was no time for that, which was equal to saying there was no time to do justice in our Courts. Magistrates and judges, as well as criminals, were subject to public opinion, and he therefore asked for the influence of the doctor, whose influence was so great, in favour of more complete investigation. There were needed a number of people of the probation-officer type—men and women with sympathy, good judgment, patience, experience, resourcefulness, and perseverance—to go into these cases and examine them. A certain amount of shame was a good thing, but it was possible to get hardened, especially if there were also a sense of injustice. There was need of something to strengthen the will power of the criminal, and it would be well if, when a person had stolen something or damaged something, he should do something to repair or make restitution.

### A Committee Appointed.

The Chairman

Said the following resolution had been sent up by Mr. Jennings: "That it be a recommendation to the Council of the British Medical Association to appoint a committee of members of the Association, with power to co-opt others not members of the medical profession, to consider the present state of the law with regard to legal responsibility for crime, and to issue a report with recommendations to the Council."

This was unanimously agreed to.

Professor Benjamin Moore, M.A., D.Sc., F.R.S. (Liverpool University),

Desired to make the point that crime was a progressive factor in our social system, and that we could not get on without crime. If it were otherwise, our laws must be perfect. It had just been said by Dr. Mercier that it was better to obey bad laws than to break good ones, but he thought that depended on the amount of breaking. If our forefathers had broken no laws, we should still be in the condition that they were in two or three hundred years ago. If injustice was done and people would not listen to protests, it became necessary to break laws in order to carry reforms through. He was a bad doctor who always gave anodynes to remove pain. It was necessary to understand crime in order to obtain a grip of social problems. Crime provided a stimulus to the work of putting social conditions right. What good was done, for instance, by sending a man who neglected his children to prison for a month? If it was necessary to deprive people of their liberty, it should be done under the pleasantest possible conditions. If we had perfect laws it would mean we were no longer progressive. Old crimes and methods of punishment were now disappearing, but new ones were arising, and he trusted they always would, because they ultimately meant still further improvement.

### Other Speakers.

Dr. E. S. Pasmore (Croydon Mental Hospital)

Said he wished to speak of crime from the mental point of view. He did not agree that all habitual criminals were defective mentally; but he thought there should be associated with the judge trying a criminal case a medical assessor, who had had experience of mental conditions, especially in asylums. He related some cases in support of his contention. He agreed with Dr. Mercier that in the case of some criminals the tendency to crime was inborn in them, and it was the duty of the State to protect society from them. In this respect the Mental Deficiency Bill now before Parliament would serve a very useful purpose, and he was not an advocate of lessened punishment for crime, as he believed in its deterrent effect.

Miss Allen

Said she had worked a good deal among prison girls, and she had been a suffragette prisoner in Holloway herself. Often the girl who came back to prison a second or third time was found to be mentally defective, the outstanding feature of which was weakness of will. Prison life did absolutely no good for them. Girls who had been to prison had told her they developed a great desire for one of three things—coffee, alcohol, vinegar. While she was a prisoner in Holloway she lay awake in the mornings between three and six longing for "Green Chartreuse," but on her release she did not indulge, fearing the formation of the habit, but she took frequent helpings of strong coffee. The condition must be worse in the case of those who were in very poor health when they went in. What was the use of sending to prison a woman who had made an attempt on her life? Prisons were capable of only forming criminals.

Dr. Buist (Dundee)

Considered that Dr. Mercier's epigrammatic style led one from axiomatic premises by incontrovertibly sound logic to faulty conclusions. He regretted more had not been said of the relationship between disease and crime, for certainly the connection was a very close one. Often what was admitted to be a disease was treated as if it were a crime. Both he regarded as expressions of a biological law, conditioned by the circumstances in which the individual was placed.

Dr. Bishop (barrister)

Said he thought Dr. Mercier's thesis might be extended to the statement that crime was the necessary outcome of the development of the social order; it was both an ethical and a physical consequence. Crime of some kind or degree would always be present so long as we had a social order; and we did not try to eradicate crime, but to limit its excesses and bring them, as much as possible, to one level. Special efforts should be devoted to improving the health of the community, and hence to bettering the race, and punishment should be part of the means employed, but should be in harmony with the conditions of the social life. Criminal law was not so much the groundwork for research by the lawyer as by the economics and the doctor and the psychiatrist. And the administration of the criminal law should not be the exclusive privilege of the lawyers, unless their training included economics and psychology. Criminal law was a code laid down without consideration of cause and effect and without considering the individual. Codes of law were survivals of the time when physical force ruled.

Mr. Dixon Kingham (barrister)

Agreed that the modern object of punishment was the protection of society, and that was exemplified in the Prevention of Crimes Act. But society should recognise some duty towards the criminal, hence the reform of the latter should have some place in the procedure. It was extraordinary that the one portal of admission to the benefits of the Borstal system was the commission of a crime, and that had been well travestied in a powerful play, and constituted a piece of keen social criticism. The criminal should not be a burden on society, and prisons should largely be made self-supporting; it had been done in Switzerland for twenty years.

Dr. Oppenheimer

Contended that the only way of solving this question intelligently was the historical one. He claimed that our ideas of punishment retained the principle of retaliation, but its primary purpose was the protection of society.

Dr. Mercier

Very briefly replied on the discussion for himself and other introducers.

